# Multi-objective location-routing optimization of first-mile pre-cooling distribution center networks in the agricultural cold chain

**DOI:** 10.1371/journal.pone.0350268

**Published:** 2026-05-29

**Authors:** Chen Ying, Yong Jin Kim

**Affiliations:** 1 School of Transportation and Logistics Engineering, Shandong Jiaotong University, Jinan, China; 2 Asia Pacific School of Logistics, Inha University, Incheon, Korea; North-Caucasus Federal University - Pyatigorsk Campus: Severo-Kavkazskij federal'nyj universitet Patigorskij institut filial, RUSSIAN FEDERATION

## Abstract

China is both a major producer and consumer of fresh agricultural products, making cold chain logistics essential for preserving quality and reducing post-harvest loss. However, insufficient pre-cooling capacity in production areas often leads to significant quality deterioration during the first-mile stage, which has not been fully addressed in existing cold chain network design studies. To bridge this gap, this study proposes an integrated optimization framework for designing a first-mile pre-cooling distribution center (DC) network. A multi-objective nonlinear mathematical model is developed to simultaneously minimize total logistics cost and maximize product freshness. To better characterize perishability, a stage-specific freshness decay function captures the nonlinear deterioration of products before and after pre-cooling. Transportation-related carbon emissions are also incorporated to enhance environmental relevance. Given the complexity of the location-routing problem, a genetic algorithm (GA) is used to obtain Pareto-optimal solutions. An empirical case study in Shandong Province, China, is conducted under three scenarios: (1) no pre-cooling, (2) decentralized pre-cooling at origins, and (3) centralized pre-cooling at regional DCs. Results show that the centralized strategy achieves superior performance, reducing total daily cost by 3.79% and producing the lowest freshness loss compared with the no-pre-cooling baseline. In contrast, decentralized origin-side pre-cooling improves freshness preservation but increases total cost by 5.27% due to higher equipment investment and weaker route efficiency. These findings demonstrate that an integrated location-routing perspective can provide more effective first-mile cold chain planning than treating pre-cooling as an isolated facility decision.

## Introduction

Fresh agricultural products are highly perishable and sensitive to temperature fluctuations during harvesting, handling, and transportation. In agro-food supply chains, quality deterioration often begins immediately after harvest, and insufficient cooling in the first mile may result in substantial freshness loss, economic waste, and reduced market value. With the continuous improvement of living standards and the rapid growth of demand for safe and high-quality fresh products, enhancing the operational efficiency of first-mile cold chain logistics has become an important issue for both academia and practice.

Among the key first-mile operations, pre-cooling plays a fundamental role in slowing physiological and biochemical deterioration before long-distance transportation and storage. Compared with direct transport without temperature control, effective pre-cooling can significantly preserve freshness, reduce spoilage, and improve the stability of subsequent cold chain processes [1]. However, in many agricultural production areas, pre-cooling infrastructure remains insufficient, unevenly distributed, or poorly coordinated with transportation activities. As a result, decision makers must determine not only where pre-cooling distribution centers should be located, but also how products should be transported from planting bases to pre-cooling centers and then to destination warehouses in an efficient and sustainable manner.

This decision problem is inherently complex for several reasons. First, facility location and route planning are strongly interdependent. The location of pre-cooling distribution centers affects service scope, routing structure, and transportation efficiency, while route design directly influences logistics cost and quality preservation. Second, freshness deterioration is dynamic and stage-dependent. In practice, the deterioration rate before pre-cooling is usually much higher than that after pre-cooling, yet such differences are often simplified in conventional optimization models. Third, under the growing emphasis on green logistics, cold chain network design should consider not only economic cost and freshness preservation but also transportation-related carbon emissions.

Although previous studies have contributed to cold chain network design, perishable logistics optimization, and low-carbon distribution planning, limited attention has been paid to the integrated design of first-mile pre-cooling distribution center networks under stage-specific freshness decay. In particular, there remains a need for a unified optimization framework that can jointly evaluate facility location, route planning, product freshness, total logistics cost, and carbon emissions while comparing alternative first-mile pre-cooling strategies.

To address this issue, this study develops a multi-objective location-routing optimization model for the design of agro-food cold chain pre-cooling distribution center networks. A stage-specific freshness decay function is proposed to distinguish the nonlinear deterioration processes before and after pre-cooling. Transportation-related carbon emissions are incorporated into the model together with logistics cost and freshness objectives. A genetic algorithm is designed to solve the resulting multi-objective nonlinear combinatorial optimization problem. Furthermore, three representative operational scenarios are compared to identify the most effective first-mile pre-cooling strategy.

The main novelties and contributions of this study are as follows:

- An integrated first-mile location-routing optimization framework is developed for agro-food cold chain networks with pre-cooling decisions.
- A stage-specific freshness decay function is proposed to distinguish the nonlinear deterioration process before and after pre-cooling.
- Transportation-related carbon emissions are incorporated into the optimization framework to improve environmental relevance.
- A genetic algorithm is designed to solve the resulting multi-objective nonlinear combinatorial optimization problem.
- Three representative operational scenarios are compared to identify the most effective first-mile pre-cooling strategy.

## Literature review

To provide a clearer overview of the existing literature and to position the present study more explicitly, representative studies on cold chain network design, perishable product logistics, and sustainability-oriented optimization are reviewed from the perspectives of facility location theory, cold chain logistics network design, pre-cooling facility planning, and integrated low-carbon optimization.

Facility location theory provides the conceptual foundation for logistics network design. Weber first established the analytical basis for location decision-making, laying the groundwork for subsequent studies on distribution center siting [2]. However, early facility location theory mainly focused on spatial cost minimization and did not consider the special operational characteristics of perishable products, such as freshness decay, cold chain requirements, or route coordination.

With the rapid development of fresh and cold chain logistics, increasing attention has been paid to the design of logistics networks for perishable agricultural products. Kuznietsov and Gromov showed that cluster-based organization can improve the efficiency of local food logistics [3]. Bortolini et al. investigated sustainable fresh food distribution through a three-objective optimization model that simultaneously considered cost, delivery time, and carbon footprint, revealing the trade-offs among economic and environmental goals [4]. Zhang Z. et al. addressed the location and scheduling of multiple units for time-spatially distributed harvest sites by proposing a mixed-integer nonlinear program and a genetic algorithm integrated with an iterative heuristic, thereby reducing total costs and quality loss compared to fixed-location facilities [5]. Dou et al. proposed a hybrid algorithm for the cold chain logistics distribution center location problem and improved computational efficiency in solving location decisions [6]. Zhang et al. further explored multipath cold-chain logistics network optimization and showed that network flexibility can improve logistics performance [7].

In recent years, researchers have increasingly recognized that cold chain network design for perishable products should account not only for cost efficiency but also for product deterioration and supply chain coordination. Singh et al. examined cold chain configuration design by incorporating location-allocation decisions, value deterioration, and data approximation, thereby extending conventional logistics planning to more realistic perishable-product contexts [8]. Ma et al. investigated the perishable food location-routing problem from a multi-agent perspective and addressed coordination and conflict in integrated logistics decisions [9]. Rabe M. et al. further considered multi-period logistics facility location under uncertain demand conditions, enriching the methodological basis for dynamic network design [10]. Nevertheless, these studies did not explicitly focus on first-mile pre-cooling distribution systems.

Pre-cooling has gradually been recognized as a critical intervention for reducing first-mile quality loss in agro-food supply chains. Ma and Wang examined the optimal location and capacity of pre-cooling facilities by considering the first-mile loss of fresh agricultural products and confirmed that pre-cooling infrastructure can significantly reduce origin-side quality deterioration [11]. However, their study focused primarily on facility location and capacity allocation, without integrating routing decisions into the optimization framework. More recent studies have further extended this line of inquiry toward more realistic and collaborative decision settings. Li and Hu investigated the site selection layout of multiple dairy distribution centers in a three-level cold chain network while jointly considering the number, location, and capacity of facilities [12]. By combining the elbow method with an improved adaptive genetic algorithm, they demonstrated that the construction quantity of distribution centers is a key factor affecting total cost and network efficiency. Zhang et al. developed a bi-objective location-routing optimization model for pre-cooling facilities considering pre-cooling delay time and time-window constraints, with the objectives of minimizing total operating cost and maximum pre-cooling delay [13]. Their findings showed that delay-sensitive decision-making is essential for origin-side pre-cooling systems, and that facility number and capacity configuration significantly affect the trade-off between cost and operational timeliness.

At the same time, the growing emphasis on sustainable logistics has encouraged researchers to incorporate low-carbon considerations and service performance into cold chain network design. Li et al. studied the location selection of low-carbon fresh fruit distribution centers by jointly considering customer satisfaction and route optimization, thereby linking network planning with environmental objectives [14]. Gong et al. constructed a bi-objective location-routing model for flower-origin pre-cooling stations from a low-carbon perspective, jointly minimizing comprehensive total cost and maximizing pre-cooling service quality [15]. By introducing carbon emission cost, freshness loss, and a gradual coverage function for service quality quantification, their study revealed the non-dominated trade-offs among economic efficiency, service performance, and environmental sustainability. This stream of research shows that cold chain logistics optimization is increasingly moving beyond single-objective cost minimization toward multi-objective coordinated decision-making.

Overall, the existing literature has generated valuable insights into cold chain logistics center location, sustainable distribution, pre-cooling facility planning, and integrated optimization. Recent studies have further enriched the field by addressing pre-cooling delay [13], low-carbon and service-quality-oriented location-routing design [15], and multi-distribution-center layout under construction quantity constraints [12]. Nevertheless, several important gaps remain. First, although freshness preservation is widely acknowledged as a key objective in cold chain logistics, most studies treat freshness deterioration as a uniform process and fail to distinguish the nonlinear decay characteristics before and after pre-cooling. Second, while carbon-related factors have been introduced into some location-routing models, transportation-related carbon emissions are still insufficiently integrated into the design of first-mile pre-cooling distribution center networks. Third, limited research has systematically compared alternative organizational strategies for first-mile pre-cooling operations within a unified optimization framework.

The existing literature are summarized in Table 1. The table highlights their main research focus, methodological features, and key findings, while also showing the specific gap addressed by this study.

**Table 1:** Summary of representative studies related to cold chain network design.

Author	Year	Focus	Method	Key findings	Research gap
Weber [2]	1922	Facility location theory	Analytical location theory	Provided foundational theory for location decisions	Did not address perishability or routing
Kuznietsov K A, Gromov V A [3]	2017	Cluster-based local food logistics	Case study analysis	Showed that clustered organization can improve local food logistics efficiency	Did not address cold chain pre-cooling or optimization modeling
Bortolini et al. [4]	2016	Fresh food sustainable distribution	Three-objective optimization	Showed trade-offs among cost, time, and carbon footprint	No pre-cooling integration
Zhang Z. [5]	2025	The location and scheduling of multiple mobile pre-cooling stations	A mixed-integer nonlinear program	Mobile pre-cooling stations can reduce the total cost	Limited freshness modeling
Singh et al. [8]	2018	Cold chain configuration design	Location-allocation modeling	Incorporated coordination, value deterioration, and data approximation into cold chain design	Did not focus on first-mile pre-cooling distribution
Dou et al. [6]	2020	Distribution center location	Hybrid algorithm	Improved solution efficiency for logistics center location	No explicit freshness-pre-cooling linkage
Ma et al. [9]	2020	Perishable food location-routing problem	Multi-agent optimization	Addressed conflict and coordination in perishable food location-routing decisions	Did not consider pre-cooling facility design
Rabe M et al. [10]	2021	Multi-period facility location	Simulation-optimization	Considered demand uncertainty in logistics network design	Not specific to first-mile cold chain systems
Li et al. [1 4]	2021	Low-carbon fresh distribution	Location-routing optimization	Linked routing decisions to carbon reduction	No pre-cooling process integration
Zhang et al. [7]	2023	Multipath cold-chain network	Network optimization	Improved flexibility and logistics efficiency	No stage-specific freshness modeling
Ma and Wang [11]	2024	Pre-cooling facility location and capacity	Optimization model	Confirmed the value of pre-cooling in reducing first-mile loss	Did not integrate routing decisions
Zhang et al. [13]	2025	Pre-cooling facility location-routing optimization	Bi-objective optimization with improved NSGA-II	Highlighted the importance of pre-cooling delay and time-window constraints in first-mile decision-making	Did not distinguish freshness decay before and after pre-cooling
Gong et al. [15]	2025	Low-carbon pre-cooling station location-routing	Bi-objective optimization with NSGA-II	Demonstrated trade-offs among cost, carbon emissions, and service quality	Focused on low-carbon service optimization rather than stage-specific freshness deterioration

### Problem description and methodology

**Fig 1:**
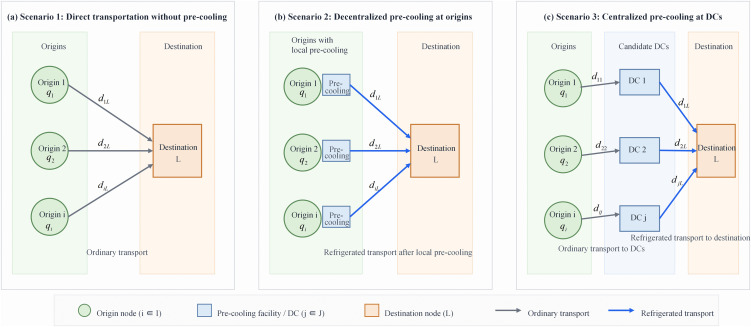
First-mile cold chain network structures under three pre-cooling scenarios. (a) Scenario 1: direct transportation without pre-cooling. (b) Scenario 2: decentralized pre-cooling at origins. (c) Scenario 3: centralized pre-cooling at candidate distribution centers (DCs). Grey arrows indicate ordinary transportation and blue arrows indicate refrigerated transportation. Source: developed by the authors.

This study considers a first-mile cold chain network for fresh agricultural products consisting of production origins, candidate pre-cooling distribution centers (DCs), and a final destination warehouse, as shown in Fig 1. The network design problem involves two interrelated decisions:

(a) determining the number and location of pre-cooling DCs; and (b) identifying efficient transportation routes between origins, DCs, and the destination.

To evaluate the operational effects of different first-mile cooling strategies, three scenarios are considered in this study.

Scenario 1 represents direct transportation without pre-cooling, in which agricultural products are shipped from planting bases to the destination without any cooling treatment during the first-mile stage.

Scenario 2 represents decentralized pre-cooling, in which products are pre-cooled near the planting bases before being transported to the destination.

Scenario 3 represents centralized pre-cooling, in which products are first collected at selected pre-cooling distribution centers, treated there, and then transported to the destination through the cold chain.

These three scenarios allow a systematic comparison of cost performance, freshness preservation, and environmental impact under different network configurations.

In addition to economic cost, this study explicitly considers product freshness and transportation-related carbon emissions. Therefore, an optimal mathematical model is proposed with the dual objectives of minimizing total cold chain cost and maximizing product freshness.

The proposed problem is a multi-objective nonlinear combinatorial optimization problem that integrates facility location, route planning, product freshness decay, and transportation-related carbon emissions. As the number of production origins, candidate distribution centers, and possible routes increases, the feasible solution space expands rapidly, making exact solution methods computationally difficult for realistic instances. Therefore, a genetic algorithm is adopted in this study because it is suitable for solving complex location-routing problems and can efficiently search for high-quality Pareto-optimal solutions under multiple conflicting objectives.

### Research framework

**Fig 2:**
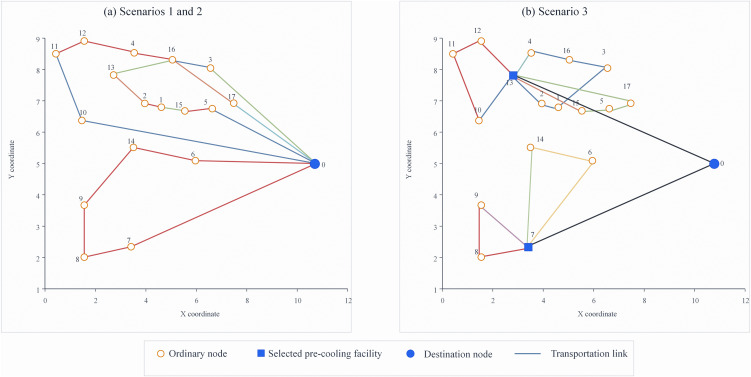
Research flowchart of the proposed first-mile pre-cooling distribution center network optimization methodology.

The overall research procedure of this study is illustrated in Fig 2. The framework starts with data collection and preprocessing, followed by scenario definition, assumption setting, and mathematical model development. Supporting analytical modules, including freshness modeling and transportation carbon-emission accounting, are then incorporated into the optimization framework. Finally, a genetic algorithm is employed to generate Pareto-optimal solutions, and the results are compared across scenarios to identify the most effective first-mile pre-cooling strategy.

The framework includes data collection, scenario definition, model assumptions, mathematical formulation, supporting analytical modules, genetic algorithm design, Pareto-solution generation, and scenario comparison. Source: developed by the authors.

The carbon-emission accounting approach used in this study is adapted from the greenhouse gas accounting standard and relevant transportation emission studies [16,17]. The freshness modeling framework is conceptually inspired by Bortolini et al., while the stage-specific nonlinear decay formulation is further developed in this study for the first-mile pre-cooling context [4].

### Model formulation

#### Assumptions.

To make the problem tractable, the following assumptions are adopted:

(1) The logistics network is structured as a two-echelon system comprising a pre-cooling distribution center (DC) and a destination DC. The pre-cooling DC operates under a known maximum capacity constraint, while the destination DC is treated as incapacitated.
(2) The locations of planting bases are predetermined, and a pre-cooling DC is to be established in proximity to these bases. Given the negligible distance between planting bases and candidate pre-cooling DC locations, the candidate DC sites are co-located with the planting bases.
(3) Multiple planting bases may be assigned to a single pre-cooling DC, but each planting base is exclusively served by one pre-cooling DC.
(4) The fleet of regular and refrigerated trucks at the pre-cooling DC is homogeneous in capacity and composition. Carbon emission coefficients for both vehicle types are known. Each vehicle departs from the pre-cooling DC, visits one or more planting bases along a designated route, and returns to the pre-cooling DC after completing loading.
(5 The location of the destination DC is known. All distances are estimated using straight-line measurements, and vehicles are assumed to maintain a constant speed.
(6) The first-mile transportation time between any planting base and its assigned pre-cooling DC shall not exceed 30 minutes.
(7) In the calculation of transportation time, it is assumed that the time for loading, unloading and transfer operations can be disregarded. The transportation time t is solely determined by the driving time.
(8) The spoilage rate of fresh agricultural products before and after pre-cooling is known and constant. Product loss within the pre-cooling DC is not considered.
(9) Product variety is not modeled; all agricultural products are assumed to have uniform unit price and pre-cooling temperature requirements.
(10) The fixed costs of the pre-cooling DC, pre-cooling operation costs, and vehicle rental costs are known. Depreciation costs are excluded from the analysis.
(11) Carbon emissions accounted for in this study are limited to those generated during transportation; emissions from pre-cooling DC operations are not included.

### Nomenclature

For clarity, the main sets, parameters, and decision variables used in the model are summarized in Table 2–4. All cost-related parameters are in Chinese CNY.

**Table 2:** Sets and indices.

Symbol	Description
I	Index of origin, i ϵ I
J	Index of candidate, j ϵ J
V	Index of normal trucks
K	Index of refrigerated trucks
S	Scenario index

**Table 3:** Decision variables.

Symbol	Description
$X_{i}$	1 if origin i is selected, 0 otherwise
$X_{j}$	1 if origin i is selected, 0 otherwise
$Y_{ij}$	1 if origin i is assigned to DC j, 0 otherwise
$Y_{iL}$	1 if origin i is assigned to destination L, 0 otherwise
$x_{ij}$	Quantity transported from origin i to DC j
$x_{jL}$	Quantity transported from DC j to destination L
$x_{iL}$	Quantity of agricultural products transported from origin i to destination L
$B_{j}$	Capacity of the DC established at location j

**Table 4:** Parameters.

Symbol	Description	Unit
$q_{i}$	Supply quantity at origin i	ton
$d_{ij}$	Distance from origin i to DC j	km
$d_{jL}$	Distance from DC j to destination L	km
$d_{iL}$	Distance from origin i to destination L	km
$c_{v}$,$c_{k}$	Unit transport cost for normal truck v and refrigerated truck k	CNY/ton
$f_{j}$	Fixed operating cost of DC j	CNY
$c_{i}$	Pre-cooling cost per unit when using portable pre-cooling equipment	CNY
$c_{j}$	Pre-cooling cost per unit at distribution center j	CNY
$Q_{v}$,$Q_{k}$	Capacity of truck v and vehicle k	ton
$h_{L}$	Storage capacity of destination DC L	ton
$\alpha_{\text{1}}$	Freshness decay coefficient before pre-cooling	$h^{-1}$
$\alpha_{\text{2}}$	Freshness decay coefficient after pre-cooling	$h^{-1}$
$t_{ij}$	Travel time from i to j	h
$e_{v}$,$e_{k}$	Carbon emission factor of vehicle v and k	kg $CO_{\text{2}}$/(ton.100 km)
μ	Carbon emissions cost per unit for vehicle	CNY/kg
$m_{p}$	Purchase cost of pre-cooling equipment	CNY
$n_{v}$,$n_{k}$	Number of normal trucks v and refrigerated trucks k deployed	unit
$uc_{v}$,$uc_{k}$	Fixed transportation cost for a normal truck v and refrigerate truck k	CNY
P	Unit value of fresh agricultural products	CNY

### Total cost minimization

Based on the above assumptions, parameter definitions, and analytical modules, a multi-objective nonlinear optimization model is formulated to determine the optimal first-mile pre-cooling distribution center network. The model jointly optimizes facility location and routing decisions while minimizing total logistics cost and maximizing product freshness. Transportation-related carbon emissions are also quantified to support comparative evaluation of different operational scenarios, the total cost can be written as:


$$\begin{array}{c} min\;Z=C_{Transport}+C_{Fix}+C_{Emission}+C_{Freshness} \end{array}$$

This study conducted separate modeling for the minimum total cost in three different scenarios.

### Freshness maximization

The second objective is to maximize the freshness of agricultural products delivered to the destination. Inspired by freshness-related distribution modeling in Bortolini et al. [4], freshness is expressed as a function of travel and handling time:


$$\begin{array}{c} min\;C_{Freshness}=\sum_{i\in I}F_{i} \end{array}$$ (1)

Where $F_{i}$ denotes the remaining freshness of the product from origin i upon arrival at the destination.

Bortolini et al. (2015) established a key framework for the multi – objective optimization of perishable agricultural product supply chains. The piecewise linear quality function they proposed correlates transportation time with product shelf – life, providing a core methodology for quantifying the freshness loss in the distribution network [4]. This framework is particularly suitable for evaluating the macro – impact of transportation time on the quality of agricultural products from the origin to the distribution center. However, in the actual circulation process, the spoilage process of agricultural products exhibits distinct stage – specific characteristics. In the first stage (T1), the products are in a period of stable quality, maintaining good appearance and edible value. As the transportation and storage time extends to the second stage (T2), the product quality begins to decline significantly, and the spoilage rate accelerates notably. By the third stage (T3), the products are completely spoiled, unfit for consumption and may even pose a hazard to human health. Therefore, although the piecewise linear model can reflect the overall downward trend of quality over time, it is difficult to accurately depict the dynamic transition process of different spoilage stages. Introducing a quality decay model with stage – specific characteristics will better conform to the non – linear spoilage law of agricultural products in actual circulation.

**Fig 3:**
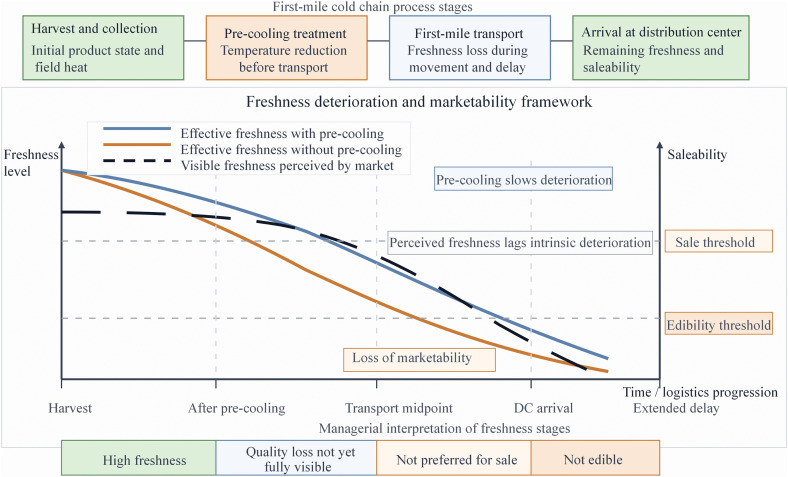
Conceptual framework of stage-specific freshness deterioration and marketability in first-mile cold chain logistics.

In the research on the supply chain of perishable agricultural products, freshness is typically modeled as a monotonically decreasing function over the transportation time, indicating that the freshness of agricultural products will continuously decline as the transportation time prolongs as illustrated in Fig 3.

The figure illustrates the difference between effective freshness trajectories with and without pre-cooling, the lag between intrinsic freshness loss and market-perceived freshness, and the saleability thresholds across logistics stages. Source: developed by the authors based on the freshness deterioration concept in [18].

For the first mile from the production site to the distribution center, considering the characteristics of post-harvest cooling treatment and short-term transportation in different scenarios, this study, based on the above theories, represents the relationship between freshness and time as a monotonically decreasing exponential curve to more accurately describe the nonlinear dynamic of quality decay. At the same time, referring to the data on the freshness loss rate of agricultural products under different temperatures released by the Zhejiang Provincial Development and Reform Commission, the freshness loss cost is quantified as the product of the freshness loss rate, the unit price of agricultural products, and the transportation volume, thereby achieving a more refined balance of spoilage costs and transportation strategies in the integrated optimization of production site selection and primary distribution routes.

To characterize perishability more accurately, a stage-specific freshness decay formulation is introduced in this study. Unlike a single uniform decay rate, the proposed formulation distinguishes the faster deterioration stage before pre-cooling from the slower deterioration stage after pre-cooling. This modeling idea is inspired by existing freshness-loss studies on perishable food logistics and adapted to the operational context of first-mile pre-cooling distribution networks considered in this paper [12].

For the stage before pre-cooling:


$$\begin{array}{c} \overset{\left(1\right)}{\underset{i}{F}}=exp(-\alpha_{\text{1}}\overset{\left(1\right)}{\underset{i}{t}}) \end{array}$$ (2)

For the stage after pre-cooling:


$$\begin{array}{c} \overset{\left(2\right)}{\underset{i}{F}}=exp(-\alpha_{\text{2}}\overset{\left(2\right)}{\underset{i}{t}}) \end{array}$$ (3)

Thus, the total freshness is:


$$\begin{array}{c} F_{i}=\overset{\left(1\right)}{\underset{i}{F}}\times \overset{\left(2\right)}{\underset{i}{F}} \end{array}$$ (4)

where α is the corrosion rate coefficient (unit: $h^{-1}$), which depends on the product and temperature condition. tdenotes the cumulative transportation time, with the unit of hours (h). $\alpha_{\text{1}}>\alpha_{\text{2}}$, reflecting that product deterioration is faster before pre-cooling and slower after pre-cooling.

### Carbon emission accounting

To accurately quantify the carbon emissions generated during the transportation phase, this study employs a vehicle-kilometer-based accounting method. The transportation carbon-emission calculation follows the emission-accounting logic commonly used in logistics studies and is adapted from the greenhouse gas accounting approach and related transportation emission studies [16,17]. The total carbon emission cost is calculated by aggregating the emissions from all vehicles used in the distribution network, as shown in Equation:


$$\begin{array}{c} C_{emission}=\mu \times \sum \left[\left(e_{v}\times n_{v}\times \sum d_{ij})+(e_{k}\times n_{k}\times \sum d_{jL}\right)\right] \end{array}$$

Where:

$C_{emission}$ represents the total carbon emission cost (CNY).

In this paper, the carbon emission data of common vehicles and refrigerated vehicles are obtained from the China carbon trading network [19]. The carbon emission cost is then calculated as the product of the unit carbon emission cost, carbon emission, transportation distance, and the number of vehicles. This accounting scope is limited to well-to-wheel emissions from fuel combustion during transportation. Emissions from pre-cooling operations at the DC and vehicle manufacturing are excluded from this study, aligning with the stated assumptions.

Although carbon emissions are not set as an independent optimization objective in the current model, they are included as an important evaluation indicator for comparing different scenarios.

To effectively solve the facility location-vehicle routing collaborative optimization model constructed in this study, the Genetic Algorithm(GA) is adopted as the core solution method. This algorithm, by simulating natural selection and genetic mechanisms, can efficiently search for approximate optimal solutions in the complex multi-objective and nonlinear constraint space, and is particularly suitable for handling combinatorial optimization problems in large-scale distribution networks.

### Model construction

The following integrated objective function is formulated by the authors for the present first-mile pre-cooling network design problem.

Scenario 1: No pre-cooling


$$\begin{array}{c} min\;Z_{\text{1}}=\sum_{i\in I}\sum_{j\in J}c_{v}d_{iL}x_{iL}+n_{v}uc_{v}+\mu \sum_{i\in I}\sum_{j\in J}e_{v}d_{iL}n_{v}+\sum_{i\in I}\sum_{j\in J}Px_{iL}Y_{iL}[1-e^{-\alpha_{\text{1}}t_{iL}}] \end{array}$$ (5)

$$\begin{array}{c} \begin{array}{c} s.t.\\ x_{iL}\leq \alpha_{\text{1}},\forall i \end{array} \end{array}$$ (6)

$$\begin{array}{c} x_{iL}\leq n_{v}Q_{v},\forall i \end{array}$$ (7)

$$\begin{array}{c} Y_{iL}\in \left\{0,1\right\},\forall i \end{array}$$ (8)

$$\begin{array}{c} x_{iL}\geq 0,\forall i \end{array}$$ (9)

Equation (5) represents the objective function, which minimizes the total cost under the no pre-cooling scenario. Constraint (6) ensures that the quantity of agro-products transported from planting base i to the destination distribution center L does not exceed the available supply at planting base i. Constraint (7) imposes the vehicle capacity limits on the transportation between planting base i and the destination distribution center L. Constraint (8) defines the binary nature of the decision variables. Constraint (9) imposes non-negativity conditions on the relevant continuous variables.

Scenario 2: Origin-side pre-cooling


$$\begin{array}{c} \begin{array}{c} min\;Z_{\text{2}}=\sum_{i\in I}\sum_{j\in J}c_{k}d_{iL}x_{iL}+n_{k}uc_{k}+\sum_{i\in I}m_{p}+\sum_{i\in I}c_{i}x_{iL}\\ +\mu \sum_{i\in I}\sum_{j\in J}e_{k}d_{iL}n_{k}+\sum_{i\in I}\sum_{j\in J}Px_{iL}Y_{iL}[1-e^{-\alpha_{\text{2}}t_{iL}}] \end{array} \end{array}$$ (10)

$$\begin{array}{c} \begin{array}{c} s.t.\\ x_{iL}\leq \alpha_{\text{2}},\forall i \end{array} \end{array}$$ (11)

$$\begin{array}{c} x_{iL}\leq n_{k}Q_{k},\forall i \end{array}$$ (12)

$$\begin{array}{c} X_{i}\in \left\{0,1\right\},\forall i \end{array}$$ (13)

$$\begin{array}{c} Y_{iL}\in \left\{0,1\right\},\forall i \end{array}$$ (14)

$$\begin{array}{c} x_{iL}\geq 0,\forall i \end{array}$$ (15)

Equation (10) defines the objective function, which represents the total cost in the pre-cooling scenario at each origin (planting base) of agricultural products. Constraint (11) ensures that the quantity of agro-products transported from planting base i to the destination distribution center L does not exceed the available supply at planting base i. Constraint (12) imposes vehicle capacity limits on the transportation from planting base i to the destination distribution center L. Constraints (13)–(14) define binary decision variables. Constraint (15) enforces non-negativity on the relevant continuous variables.

Scenario 3: Centralized DC pre-cooling


$$\begin{array}{c} \begin{array}{c} min\;Z_{\text{3}}=\sum_{j\in J}f_{j}X_{j}+\sum_{i\in I}\sum_{j\in J}c_{j}x_{ij}+\sum_{i\in I}\sum_{j\in J}c_{v}d_{ij}x_{ij}\\ +\mu \sum_{i\in I}\sum_{j\in J}e_{v}d_{ij}n_{v}+\mu \sum_{i\in I}\sum_{j\in J}e_{k}d_{jL}n_{k}+n_{v}uc_{v}+n_{k}uc_{k}\\ +\sum_{i\in I}\sum_{j\in J}c_{k}d_{jL}x_{jL}+\sum_{i\in I}\sum_{j\in J}Px_{ij}Y_{ij}[1-e^{-\alpha_{\text{1}}t_{ij}}]+\sum_{i\in I}\sum_{j\in J}Px_{jL}[1-e^{-\alpha_{\text{2}}t_{jL}}] \end{array} \end{array}$$ (16)

$$\begin{array}{c} \begin{array}{c} s.t.\\ x_{ij}\leq \alpha_{\text{1}},\forall i,\forall j \end{array} \end{array}$$ (17)

$$\begin{array}{c} x_{ij}\leq MY_{ij},\forall i,\forall j \end{array}$$ (18)

$$\begin{array}{c} Y_{ij}\leq X_{j},\forall i,\forall j \end{array}$$ (19)

$$\begin{array}{c} x_{jL}\leq B_{j},\forall j \end{array}$$ (20)

$$\begin{array}{c} x_{jL}\leq \sum x_{ij},\forall i,\forall j \end{array}$$ (21)

$$\begin{array}{c} x_{jL}\leq X_{j},\forall j \end{array}$$ (22)

$$\begin{array}{c} \sum_{j\in J}x_{jL}\leq h_{L},\forall j \end{array}$$ (23)

$$\begin{array}{c} x_{jL}\leq n_{k}Q_{k},\forall j \end{array}$$ (24)

$$\begin{array}{c} \sum_{j\in J}Y_{ij}=1,\forall i,\forall j \end{array}$$ (25)

$$\begin{array}{c} \sum X_{j}\geq 1,\forall j \end{array}$$ (26)

$$\begin{array}{c} x_{ij}\leq n_{v}Q_{v},\forall i,\forall j \end{array}$$ (27)

$$\begin{array}{c} X_{j}\in \left\{0,1\right\},\forall j \end{array}$$ (28)

$$\begin{array}{c} Y_{ij}\in \left\{0,1\right\},\forall i,\forall j \end{array}$$ (29)

$$\begin{array}{c} x_{ij},x_{jL},x_{iL}\geq 0,\forall i,\forall j \end{array}$$ (30)

Equation (16) defines the objective function, representing the total cost associated with establishing origin-based pre-cooling distribution centers (DCs) for agricultural products. Constraint (17) ensures that the amount of agro-products transported from planting base i to DC j does not exceed the available supply at planting base i. Constraint (18) is a big-M constraint, which ensures that agro-products are transported only between assigned nodes. Constraint (19) ensures that each planting base i can only be assigned to an established DC at candidate location j. Constraint (20) restricts the quantity of agro-products transported from each DC to the destination warehouse to not exceed the DC’s storage capacity. Constraint (21) ensures that the total capacity of each DC equals the sum of supplies from the planting bases assigned to it. Constraint (22) guarantees that only established pre-cooling DCs can be assigned to the destination DC. Constraint (23) limits the total quantity of agro-products transported from all pre-cooling DCs to the destination DC to be within its storage capacity. Constraint (24) ensures that the amount transported from each pre-cooling DC to the destination DC complies with the vehicle capacity limits. Constraint (25) enforces that each planting base can supply agro-products to only one DC. Constraint (26) ensures that at least one DC is established. Constraint (27) imposes vehicle capacity restrictions between planting base iii and DC j. Constraints (28)–(29) define binary decision variables. Constraint (30) imposes non-negativity conditions on continuous decision variables.

The proposed model is formulated for optimizing the first-mile pre-cooling distribution center network for fresh agricultural products. First determines the optimal location of pre-cooling DCs, then focuses on transport cost minimization. The problem is NP-hard, traditional operations research methods are not sufficient. Therefore, considering the complexity and combinatorial nature of the network layout problem, a genetic algorithm is adopted in this study to efficiently solve the optimization model.

### Case study

This study adopts a case analysis approach to verify the practical applicability of the proposed model. The case study focuses on one representative category of fresh agricultural product in Shandong Province, namely strawberry tomatoes. This product was selected because it is a high-value variety, have garnered considerable attention due to their distinctive taste and superior flavor. But it highly perishable and requires timely pre-cooling after harvest to reduce first-mile quality deterioration. The case network, which is based in City H in Shandong Province, consists of 17 production origins (I=17), 17 candidate pre-cooling distribution centers (J=17), and one final destination warehouse.

The production origins represent major agricultural production areas, while the candidate distribution centers are selected based on regional logistics accessibility and potential pre-cooling service coverage. Product yield in this study is represented by the available supply volume at each production origin. The total yield considered in the case study is shown in Table 5, and the yield at each origin is used as an input supply parameter in the location-routing optimization model.

**Table 5:** The annual output of strawberry tomatoes in City H.

Planting base	Total annual output(ton)	Average daily output(ton)
1	3075	17
2	1568	8.7
3	2863	15.9
4	26200	145.5
5	1628	9
6	1639	9.1
7	6400	35.5
8	1679	9.3
9	2435	13.5
10	3376	18.7
11	6586	36.5
12	6144	34.1
13	16174	89.8
14	1466	8.1
15	1205	6.6
16	12916	71.7
17	640	3.5

Food quality in this study is represented by product freshness, which is treated as a normalized quality indicator ranging from 0 to 1. A higher freshness value indicates better retained quality during transportation and handling. Because detailed physicochemical quality measurements were not available for all production origins, freshness is used as the main proxy for product quality. The freshness retention ratio is calculated based on the stage-specific freshness decay function, and the final freshness at the destination reflects the quality retained after the first-mile transportation and pre-cooling process.

To improve the transparency and practical interpretability of the case study, the main operating parameters used in the model are summarized and explained in Table 6-7. These parameters include supply quantities, facility capacities, transportation costs, travel speeds, freshness decay coefficients, and carbon-emission factors. In addition to numerical values, the practical significance of each parameter is clarified to show how it affects first-mile cold chain network design and optimization outcomes.

**Table 6:** Main operating parameters used in the case study and their practical significance.

Parameter	Description	Unit	Value	Practical significance
$c_{v}$	Unit transportation cost of ordinary transportation	CNY/ton.km	1.2	Captures transport expenditure before pre-cooling or in direct shipment
$c_{k}$	Unit transportation cost of refrigerated transportation	CNY/ton.km	3	Captures transport expenditure after pre-cooling under cold-chain operation
$m_{p}$	Purchase cost of pre-cooling equipmen	CNY	140,000	Reflects the fixed equipment cost incurred when a candidate center is selected for operation
$f_{j}$	Fixed opening cost of candidate pre-cooling distribution center j	CNY	600,000	Reflects the fixed daily cost incurred when a candidate center is selected for operation
$\alpha_{\text{1}}$	Freshness decay coefficient before pre-cooling	1/h	0.25	Reflects the rapid deterioration rate of agricultural products without cooling treatment
$\alpha_{\text{2}}$	Freshness decay coefficient after pre-cooling	1/h	0.15	Reflects the slower deterioration rate after cooling treatment is applied
μ	Carbon emissions cost per unit for vehicle	CNY/kg	0.0528	Converts total vehicle carbon emissions into monetary cost
$e_{v}$	Carbon emission coefficient of ordinary transportation	kg $CO_{\text{2}}$/(ton.100 km)	29.7	Measures emissions generated during non-refrigerated transport
$e_{k}$	Carbon emission coefficient of refrigerated transportation	kg $CO_{\text{2}}$/(ton.100 km)	37.8	Measures emissions generated during refrigerated transport

**Table 7:** Road Network Distance Between Nodes (unit: km).

	L	F1	F2	F3	F4	F5	F6	F7	F8	F9	F10	F11	F12	F13	F14	F15	F16	F17
L	0	67.4	71.3	63.6	81.4	48.6	50.2	81.6	100	95.6	95.3	109.4	100.8	82.7	76.6	62.5	69.5	44.9
F1	–	0	4.0	15.8	19.0	19.4	23.7	51.8	61.6	48.5	28.6	42.4	35.4	16.1	24.5	4.9	14.4	24.9
F2	–	–	0	18.2	17.3	23.4	26.5	51.5	60.1	46.5	24.6	38.7	32.2	12.9	23.1	8.9	15.6	28.9
F3	–	–	–	0	18.2	17.2	31.8	66.4	77.2	64.3	40.0	47.9	37.9	22.6	40.2	14.6	6.1	18.9
F4	–	–	–	–	0	33.3	42.4	67.9	74.5	59.7	26.4	29.8	19.7	8.9	38.7	22.5	12.1	36.5
F5	–	–	–	–	–	0	17.9	57.6	71.6	61.1	48.0	60.8	52.4	34.1	37.1	14.6	22.2	6.3
F6	–	–	–	–	–	–	0	40.6	56.1	47.9	47.1	64.5	58.7	39.4	26.5	20.0	34.5	22.9
F7	–	–	–	–	–	–	–	0	18.7	21.8	54.2	74.8	75.3	60.3	29.4	51.8	66.2	63.3
F8	–	–	–	–	–	–	–	–	0	15.5	54.8	73.9	77.2	65.9	37.1	62.9	75.7	77.7
F9	–	–	–	–	–	–	–	–	–	0	39.3	58.5	61.7	51.0	24.4	50.5	62.0	67.4
F10	–	–	–	–	–	–	–	–	–	–	0	20.7	22.4	18.1	27.4	33.4	34.9	53.5
F11	–	–	–	–	–	–	–	–	–	–	–	0	11.6	26.7	48.0	47.2	41.8	65.1
F12	–	–	–	–	–	–	–	–	–	–	–	–	0	19.3	46.7	39.9	31.8	56.0
F13	–	–	–	–	–	–	–	–	–	–	–	–	–	0	31.0	20.7	17.0	38.6
F14	–	–	–	–	–	–	–	–	–	–	–	–	–	–	0	26.1	38.6	43.4
F15	–	–	–	–	–	–	–	–	–	–	–	–	–	–	–	0	15.3	20.3
F16	–	–	–	–	–	–	–	–	–	–	–	–	–	–	–	–	0	24.7
F17	–	–	–	–	–	–	–	–	–	–	–	–	–	–	–	–	–	0

In the context of fresh agricultural product harvesting, the harvesting and transportation processes of strawberry tomatoes are critical for maintaining product freshness. To preserve quality, strawberry tomatoes must be transported immediately after harvest, thereby maximizing the retention of their fresh state. This study obtained annual total tomato yield data from major planting bases in City H by referring to the *2020 Statistical Yearbook of City H*. Given that the harvesting cycle of tomatoes typically spans 60–90 days, with two harvest periods per year, most production bases adopt a daily batch-harvesting approach for ripe fruits, followed by immediate transportation.

To present the findings more clearly and logically, the results are organized according to the three operational scenarios. The performance of each scenario is evaluated using three main indicators: total logistics cost, product freshness at the destination, and transportation-related carbon emissions. In addition, the selected pre-cooling distribution centers, routing patterns, and total transportation distance are compared to explain the operational reasons behind the observed differences. Because this study is based on deterministic optimization outputs rather than random experimental samples, conventional statistical significance tests are not applied. Instead, scenario-based comparison, Pareto-optimal solution analysis, and percentage performance differences are used to support the interpretation of the results.

The algorithm parameters were configured as follows: the maximum number of generations (MAXGEN) was set to 300, the population size was 1000, the crossover rate (pc) was 0.90, and the mutation rate (pm) was 0.05. To ensure the robustness of the GA, a sensitivity analysis on these key parameters was performed. Varying pc between 0.85–0.95 and pm between 0.03–0.07 resulted in less than 2% variation in the final objective function value, indicating stable convergence. Similarly, increasing MAXGEN to 500 did not yield significant improvement, confirming that 300 generations were sufficient for this problem scale. The algorithm is implemented and executed using Matlab R2016b, and the calculation example is evaluated on a machine with a CPU speed of 2.8GHz and 8GB of RAM.

In the scenario 1 no pre-cooling in the production area, the results of the calculations indicate that the final objective function value (total daily cost) is 60,838.6 CNY/day. Given the vehicle capacity, there are a total of 3 general vehicles and 3 delivery routes.

The results of scenario 2, the calculations indicate that with pre-cooling at each origin of agricultural product, the final objective function value obtained (total daily cost) is 64,046 CNY/day. In the case of considering the vehicle capacity, a total of 3 refrigerated vehicles and 3 delivery routes are used.

**Fig 4:**
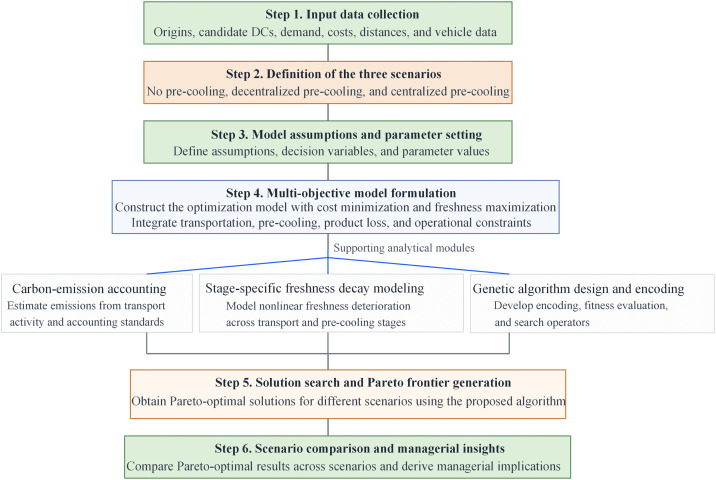
Optimal transportation networks under different pre-cooling scenarios. (a) Optimal network under Scenarios 1 and 2. (b) Optimal network under Scenario 3. Circular nodes represent ordinary origin nodes, square nodes indicate selected pre-cooling facilities, and the large blue node denotes the terminal destination. Source: developed by the authors.

The results of scenario 3, the calculations indicate that in the case of pre-cooling at the pre-cooling DC of the agricultural product origin, the final objective function value (total daily cost) is 58,521.2 CNY/day. In consideration of the vehicle capacity, 2 general vehicles, 2 refrigerated vehicles, and 5 delivery routes are utilized. As shown in Fig 4, and the resulting data is summarized in Table 8 and 9.

**Table 8:** Detailed Delivery Routes and Metrics for Three Scenarios.

Scenario Number	DC Number	Vehicle Number	Distribution route	Total Cost(CNY)
1	0	1	0- > 5- > 15- > 1- > 2- > 13- > 16- > 17- > 0	60838.6
		2	0- > 7- > 8- > 9- > 14- > 6- > 0	
		3	0- > 10- > 11- > 12- > 4- > 3- > 0	
2	0	1	0- > 7- > 8- > 9- > 14- > 6- > 0	64046
		2	0- > 10- > 11- > 12- > 4- > 16- > 0	
		3	0- > 5- > 15- > 1- > 2- > 13- > 3- > 17- > 0	
3	7 13	1	13- > 10- > 11- > 12- > 13	58521.2
		2	7- > 14- > 6- > 7	
		3	13- > 17- > 5- > 15- > 13	
		4	13- > 4- > 16- > 3- > 1- > 2- > 13	
		5	7- > 8- > 9- > 7	

**Table 9:** Comparison of Solution Results for the Three Scenarios (CNY/day).

		Scenario 1	Scenario 2	Scenario 3
Cost	Pre-cooling equipment cost	–	1304.2	657.5
	Pre-cooling cost	–	14585.4	10025.8
	Transportation cos	7542.7	15897.5	18066.1
Effect	Carbon emission cost	9.7	12.3	15.6
	Freshness loss	53286.2	32246.6	29756.2
Total Cost		60838.6	64046	58521.2

As shown in Table 9, Scenario 3 achieves the most balanced performance across cost, freshness, and carbon-emission indicators, whereas Scenario 2 emphasizes freshness preservation but suffers from higher facility investment and lower routing efficiency.

In these three scenarios, Scenarios 1 and 2 share the same routing topology because both involve direct shipment from origins to the destination, while the difference lies in the pre-cooling treatment and vehicle type.

Scenario 2 involved pre-cooling at each origin. Theoretically, this should have resulted in the lowest freshness loss. However, the results showed that Scenario 3 had the lowest freshness loss. The underlying reasons for this include the following:

1. Efficiency of pre-cooling equipment: Due to differences in pre-cooling equipment, the capacity of the equipment in Scenario 2 was limited and not fully functional. The pre-cooling process had to be conducted in stages, causing some products to still experience freshness loss. In contrast, Scenario 3 performed pre-cooling at a centralized DC, which offered greater scale and efficiency advantages. This allowed for more uniform and effective temperature control, thereby reducing freshness loss.
2. Vehicle type and transportation network: Scenario 2 used three refrigerated trucks but only had three delivery routes. Compared to Scenario 3, route optimization was less effective, resulting in longer transportation times for some products and further freshness decline. Scenario 3 utilized both regular and refrigerated trucks and implemented more delivery routes, reducing transportation time and distance for each vehicle. This optimization effectively minimized freshness loss during transit.

These findings suggest that pre-cooling should not be planned as an isolated technical activity. Instead, it should be embedded into first-mile network design in a coordinated manner. In regions where farm-level pre-cooling infrastructure is underdeveloped, strategically located centralized pre-cooling DCs may offer a more practical and cost-effective solution.

## Conclusion

This study developed a multi-objective location-routing optimization framework for the design of first-mile pre-cooling distribution center networks in the agricultural cold chain. The proposed model jointly considers facility location, route planning, total logistics cost, product freshness, and transportation-related carbon emissions. To better characterize perishability of fresh agricultural products, a stage-specific freshness decay function was introduced to distinguish deterioration before and after pre-cooling. A genetic algorithm was then designed to generate Pareto-optimal solutions under three operational scenarios in a case study of Shandong Province, China.

The key findings are summarized as follows:

1. The stage-specific freshness decay model provides a more realistic representation of first-mile quality deterioration and improves the practical relevance of the optimization results.
2. The inclusion of transportation-related carbon emissions enhances the comprehensiveness of the decision framework and supports more sustainable cold chain network planning.
3. Centralized pre-cooling at regional DCs achieves the best overall performance among the three scenarios, reducing total daily cost by 3.79% and producing the lowest freshness loss relative to the no-precooling baseline.
4. Decentralized pre-cooling at origins improves freshness preservation but is less cost-effective, increasing total cost by 5.27% because of high equipment investment and weaker route efficiency.
5. An integrated location-routing perspective is more effective than treating pre-cooling as an isolated facility decision.

From a managerial perspective, the results indicate that establishing strategically located centralized pre-cooling distribution centers can be a cost-effective and quality-preserving approach for improving the first mile of the agro-food cold chain, especially in areas where origin-side pre-cooling infrastructure is inadequate. Such a strategy can help reduce early-stage product deterioration, improve transportation efficiency, and support more sustainable cold chain network planning.

This study also has several limitations. First, the model is developed under deterministic assumptions regarding yield, travel time, and demand. Second, only transportation-related carbon emissions are considered, while operational emissions from pre-cooling facilities are not explicitly included. Third, the case study focuses on strawberry tomatoes and does not consider heterogeneous product-specific temperature requirements. Future research may extend the model by incorporating uncertainty in yield, travel time, and demand; operational emissions from pre-cooling facilities; time-window constraints; and multi-product heterogeneous cold chain settings.

## References

[1] Tian CQ. Research and Development of China’s Strategic New Industries Cold Chain Logistics. Beijing: Machinery Industry Press. 2020.

[2] Weber A. Ueber den Standort der Industrien. Tübingen: JCB Mohr (Paul Siebeck). 1922.

[3] Kuznietsov KA, Gromov VA, Skorohod VA. Cluster-based supply chain logistics: a case study of a Ukrainian food distributor. IMA J Management Math. 2016;:dpw009. doi: 10.1093/imaman/dpw009

[4] Bortolini M, Faccio M, Ferrari E, Gamberi M, Pilati F. Fresh food sustainable distribution: cost, delivery time and carbon footprint three-objective optimization. Journal of Food Engineering. 2016;174:56–67. doi: 10.1016/j.jfoodeng.2015.11.014

[5] Zhang Z, Wang L, Tan Z, Wang S. Locating and scheduling mobile pre-cooling stations for agricultural products. J Operational Res Society. 2025;77(5):1179–203. doi: 10.1080/01605682.2025.2520984

[6] Dou S, Liu G, Yang Y. A New Hybrid Algorithm for Cold Chain Logistics Distribution Center Location Problem. IEEE Access. 2020;8:88769–76. doi: 10.1109/access.2020.2990988

[7] Zhang G, Dai L, Yin X, Leng L, Chen H. Optimization of multipath cold-chain logistics network. Soft Comput. 2023;27(23):18041–59. doi: 10.1007/s00500-023-09013-y

[8] Singh AK, Subramanian N, Pawar KS, Bai R. Cold chain configuration design: location-allocation decision-making using coordination, value deterioration, and big data approximation. Int J Prod Res. 2018;56(1–2):651–71.

[9] Ma Y, Ying B, Zhou X, Wang P. Multi-agent optimization model and algorithm for perishable food location-routing problem with conflict and coordination. Syst Eng Theor Pract. 2020;40(12):3194–209.

[10] Rabe M, Gonzalez-Feliu J, Chicaiza-Vaca J, Tordecilla RD. Simulation-Optimization Approach for Multi-Period Facility Location Problems with Forecasted and Random Demands in a Last-Mile Logistics Application. Algorithms. 2021;14(2):41. doi: 10.3390/a14020041

[11] Ma Z, Wang Y. Optimal location and capacity of pre-cooling facilities considering the first-mile loss of fresh agri-products. Chin J Manag Sci. 2024;32:315–23.

[12] Li L, Hu X. Research on Cold Chain Site Selection Layout for Multiple Distribution Centers Considering Construction Quantity. Advances in Economics, Business and Management Research. Atlantis Press International BV. 2024. p. 1018–26. doi: 10.2991/978-94-6463-570-6_102

[13] Zhang SF, Xu B, Mao HJ, Yu XL. Location and route optimization of pre-cooling facilities considering pre-cooling delay time. J Southeast Univ (Nat Sci Ed). 2025;55(4):1123–30.

[14] Li Z, Fu Y, Li M. Study on Location Selection of Low-Carbon Fresh Fruit Distribution Center Considering Customer Satisfaction-Route Optimization. IOP Conf Ser: Earth Environ Sci. 2021;687(1):012179. doi: 10.1088/1755-1315/687/1/012179

[15] Gong YM, Jiao BX, Ouyang KY. Site selection and route planning of flower pre-cooling stations from a low-carbon perspective. Enterprise Innovation Management. 2025;(4):60–5.

[16] Malwe PD, Gawali B, Deshpande M, Panchal H, Darade P. Energy nexus for grapes production: A case study of Sangli region in India. Energy Nexus. 2022;8:100145. doi: 10.1016/j.nexus.2022.100145

[17] Zhang H, Xiong Y, He M, Qu C. Location model for distribution centers for fulfilling electronic orders of fresh foods under uncertain demand. Scientific Programming. 2017;2017:1–13. doi: 10.1155/2017/3423562

[18] Li QY, Long GY, Ba LJ. Effects of logistics condition on fruit quality of postharvest litchi during storage. Guangdong Agricultural Sciences. 2014;41(16):96–9.

[19] Ministry of Ecology and Environment of the People’s Republic of China. Progress Report of China’s National Carbon Market. 2025.

